# Targeting CD38 for acute leukemia

**DOI:** 10.3389/fonc.2022.1007783

**Published:** 2022-10-12

**Authors:** Xushu Zhong, Hongbing Ma

**Affiliations:** Hematology Department, West China Hospital, Sichuan University, Chengdu, China

**Keywords:** CD38mAb, bispecific antibodies, anti-CD38 CAR T, acute myeloid leukemia, acute lymphoblastic leukemia

## Abstract

Acute leukemia (AL) is a hematological malignancy, and the prognosis of most AL patients hasn’t improved significantly, particularly for relapsed or refractory (R/R) AL. Therefore, new treatments for R/R adult acute myeloid leukemia (AML) and acute lymphoblastic leukemia (ALL) are urgently necessary. Novel developments have been made in AL treatment, including target and immune therapies. CD38 is one of the targets due to its high expression in many hematological malignancies, including multiple myeloma, ALL and a subset of AML. Consequently, targeting CD38 therapies, including CD38 monoclonal antibodies (mAbs), bispecific antibodies, and CAR-T cell therapy, exhibit promising efficacy in treating multiple myeloma without significant toxicity and are being explored in other hematological malignancies and nonhematological diseases. Herein, this review focuses on targeting CD38 therapies in ALL and AML, which demonstrate sound antileukemic effects in acute leukemia and are expected to become effective treatment methods.

## Introduction

Acute leukemia (AL), including acute myeloid leukemia (AML) and acute lymphocytic leukemia (ALL), is a hematological malignancy originating from the abnormal cloning of hematopoietic stem cells. It is characterized by aberrant proliferation of immature hematopoietic cells, thereby inhibiting normal bone marrow hematopoietic function. The prognosis of most AML patients has not improved significantly owing to its heterogeneity. Indeed, its five-year overall survival rate is less than 40% in patients under 60 years old, with a worse prognosis in patients older than 60 years old as well as in relapsed or refractory (R/R) AML (1, 2). Similarly, the prognosis of adult ALL is poor, particularly for R/R ALL patients, with an overall survival rate of less than 7% (3, 4). Therefore, new treatments for R/R adult AML and ALL are urgently needed. Based on a better understanding of the pathogenesis and pathophysiology of AL in recent years, novel advancements have been made in AL treatment, including target and immune therapies. CD38 is one of the targets in view of its high expression in several hematological malignancies, particularly in multiple myeloma (MM), with minimal or no expression in normal tissues (5). CD38-targeting therapies, including CD38 monoclonal antibodies (mAbs), bispecific antibodies, and CAR-T cell therapy, have exhibited outstanding efficacy in treating MM without significant toxicity. They are also being explored for other hematological malignancies and have demonstrated promising outcomes. This review focuses on the utilization of targeting CD38 therapies in ALL and AML, including preclinical and ongoing clinical trials of these agents, with the goal of providing novel insights for the treatment of R/R AL.

## Structure and functions of CD38

Reinherz EL et al. discovered CD38 in 1980 while searching for T cell receptors *via* murine monoclonal antibodies. They initially thought it was a molecule that activates thymus T cells (6). Later, CD38 was revealed to be a class II transmembrane glycoprotein on the cell surface that contains a C-terminal extracellular domain, a transmembrane domain, and an N-terminal intracellular domain (7–9), acting as a tetramer on the cell surface (9).

The CD38 gene is located on chromosome 4p15 and consists of eight exons and seven introns (10, 11). The CD38 promoter region comprises a CpG island, several immunological transcription factor binding sites located upstream of the CpG island (11), and a retinoic acid response element in the first intron (12). CD38 transcription and expression are enhanced by binding to the aforementioned sites.

The human CD38 antigen acts as a receptor (5, 8, 13, 14) and a catalytic enzyme (5, 15–18). As a receptor, it is primarily involved in cell adhesion and migration through binding with CD31 and is also related to cell activation, proliferation, and differentiation. Apart from receptor functions, CD38 exerts extracellular enzyme activities with multiple functions of cyclase and hydrolase. It uses nicotinamide adenine dinucleotide (NAD+) as a substrate to generate cyclic ADP ribose (cADPR) and ADPR and catalyzes nicotinic adenine dinucleotide phosphate (NAADP+) production from nicotinamide adenine dinucleotide phosphate (NADP+) (5, 15–18), thereby mediating Ca2+ influx (19, 20), activating various signaling pathways (20, 21), and participating in effector cell-mediated immunosuppression (22).

## CD38 expression in healthy tissues and diseases

CD38 is predominantly expressed in hematopoietic cells such as lymphocytes and myeloid cells, with a high expression in early and activated cells and a low expression in mature cells (5). Previous studies have validated that CD38 is highly expressed in plasma cells (378.4 nTPM) but moderately expressed in activated T cells (64.8 nTPM) and NK cells (79.3 nTPM) (5). In addition, it is expressed in germinal center lymphocytes, dendritic cells (33.8 nTPM), red blood cells (78.6 nTPM), and platelets (5, 23–25). Additionally, nonhematopoietic tissue cells, including Purkinje cells, neurofibrils, prostate endothelial cells (8.1 nTPM), islet β cells, retinal cells, and muscle fibroblasts of smooth and striated muscles (41.5 nTPM), express CD38 (5).

Various studies have corroborated that CD38 is expressed at different levels in various neoplastic and nonneoplastic diseases, particularly in hematological malignancies such as multiple myeloma (100% CD38+) (26), chronic lymphocytic leukemia (30-50% CD38+) (27), Waldenstrom macroglobulinemia (40% CD38+) (28), primary systemic amyloidosis (53% CD38+) (29), mantle cell lymphoma (60% CD38+) (30), T cell lymphoma (50-80% CD38+) (31), NK/T cell lymphoma (90% CD38+) (32, 33), etc.

CD38 is expressed in AML and ALL. Marinov J was the first to evaluate CD38 expression in 72 leukemia patients and noted that the positive rate of CD38 was up to 75% in myeloid tumors and acute non-T lymphoblastic leukemia (34). Afsaneh K et al. reported that 58.2% of 304 AML cases expressed CD38, whereas only 5% of acute promyelocytic leukemia (APL) cases did (35). Wang et al. conducted an immunophenotypic analysis on 109 AML patients and noted that the positive rate of CD38 was as high as 91.7% (100/109) (36). Although the positive rates of CD38 in AML reported by various studies are not low, its expression level on cell surfaces varies significantly. Naik et al. examined CD38 expression in leukemia cells from 37 AML patients and found significant differences in expression levels in these samples, ranging from 300+ to 6000+ of CD38 antigen density on leukemic blast (37). They further measured CD38 levels in AML patients upon diagnosis and after treatment with MRD; no significant difference was found. Similarly, Dos Santos et al. examined CD38 expression levels in AML cell lines (n=9) and AML patient cells (n=10) (38), and confirmed that CD38 expression significantly differed between AML cell lines (12,827 ± 19,320 molecules/cell) and AML primary cells (11,560 ± 8,175 molecules/cell). In another study of AML cell lines, CD38 expression levels significantly differed among AML cell lines (39). Indeed, the cell surface CD38 density of seven AML cell lines (HL-60, U-937, THP-1, MOLM-13, UOC-M1, Oci-AML2, and KasUMI-1) was detected, with CD38 levels ranging from very high (UOC-M1, 46.5 ± 4.9), high (OCI-AML2, 16.6 ± 1.5), low (THP-1, 7.0 ± 0.2), very low (molm-13, 2.1 ± 1.0 and Kasumi-1, 1.1 ± 1.2), to CD38-negative (HL-60, U-937).

For ALL, Afsaneh K et al. determined that the positive rate of CD38 in 138 ALL patients accounted for 78.2% of all cases (35). Immunophenotypic analysis of 282 ALL patients conducted in Iraq by Sana D. Jalal et al. revealed that 80.5% (194/241) of B-ALL patient samples and 95.1% (39/41) of T-ALL patient samples expressed CD38 (40). Jutta Deckert et al. investigated CD38 expression levels in different CD38-positive cell lines and identified that CD38 levels were significantly lower in B-ALL cells than in T-ALL cells (41). A study examining 50 B-ALL and 50 T-ALL primary cells showed that the median density of CD38 in T-ALL and B-ALL samples was 41,026 copies/cell and 28,137 copies/cell, respectively (42). According to Naik et al., CD38 expression levels were more consistent in 12 adult T-ALL samples than in 37 samples from AML patients at the same time (37). Collectively, these studies suggest that the CD38 expression level in T-ALL is more consistent than that in AML, with higher expression than that in B-ALL. Moreover, Bride et al. identified that CD38 was stably expressed in tumor cells of T-ALL patients, regardless of diagnosis, after induction chemotherapy or relapse (43). Researchers from India determined comparable results when examining CD38 expression levels in patients with T-ALL at diagnosis, after chemotherapy, in relapses, and in refractory status (44).

## CD38 mAbs

CD38 is abundantly expressed in multiple myeloma (MM) (26) and some non-Hodgkin’s lymphomas (NHLs) (27, 30, 33, 34), and is emerging as a new therapeutic target for these diseases. In addition, CD38 mAbs, including daratumumab (DARA), isatuximab, MOR202, and TAK079, have exhibited remarkable efficacy in MM.

**Figure 1:**
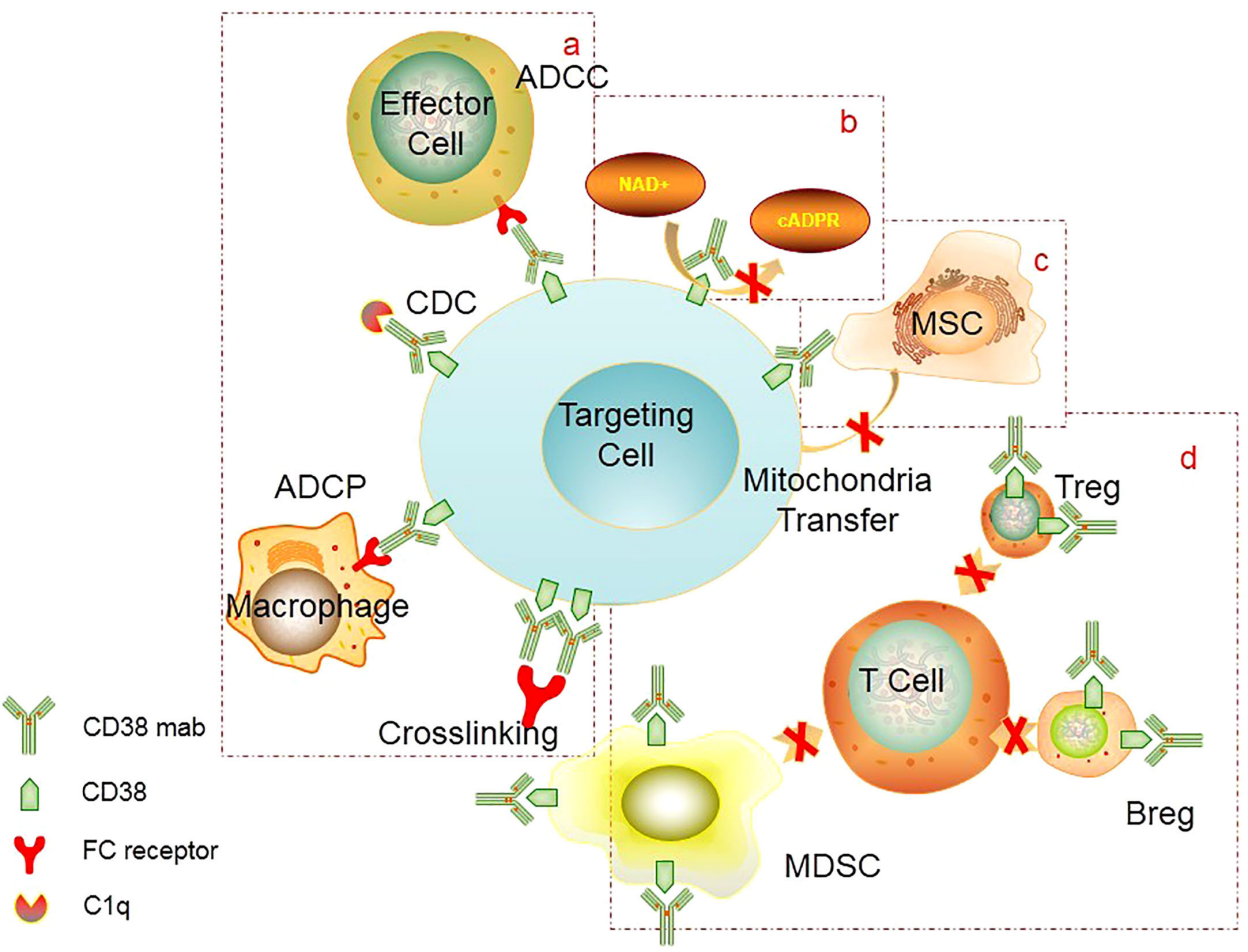
Mechanism of CD38 mAb: **(A)** FC-mediated ADCC, ADCP, cross-linking to induce apoptosis, CDC. **(B)** Inhibition of CD38 activity. CD38 mAb can block the catalytic activity of CD38 cyclase and hydrolase, thereby preventing calcium influx and inhibiting cell signal transduction. **(C)** Inhibition of mitochondrial transfer. CD38 mAb can inhibit mitochondrial transfer from mesenchymal stem cells to AL cells, thereby reducing metabolic capacity of tumor cells and ultimately inhibiting tumor proliferation. **(D)** Inhibition of immunosuppressive cells. CD38 mAb can eliminate CD38+ regulatory cells (Treg, Breg, MDSC), which can promote the expansion of effector T cells and enhance the immune killing effect. ADCC, Antibody-dependent cell-mediated cytotoxicity; ADCP, Antibody-dependent cellular phagocytosis; CDC, complement-dependent cytotoxicity; MSC, mescenchymal stem cell; MDSC, myeloid-derived suppressor cells; Treg, regulatory T cells; Breg, regulatory B cells.

Akin to other mAbs, CD38 antibodies inhibit tumor cell growth through complement-dependent cytotoxicity (CDC) (41, 45–47), antibody-dependent cytotoxicity (ADCC) (41, 45, 46), and antibody-dependent macrophage phagocytosis (ADCP) (48, 49) and induce apoptosis (45, 50). In addition, CD38 mAbs can prevent calcium influx and inhibit cell signal transduction by suppressing the catalytic activity of CD38 cyclase and hydrolase (51). It can further inhibit tumor cell growth by modulating the bone marrow microenvironment (52). (**Figure 1**) However, different monoclonal antibodies have varied action focuses. DARA is a humanized IgG1κ monoclonal antibody(mAb) that specifically binds to the epitopes of the cell surface of CD38, directly killing tumor cells through immune-mediated cytotoxicity, such as ADCC, CDC, and ADCP, and inducing apoptosis *via* FCγ receptor-mediated cross-linking (45–50). It can further inhibit the enzyme activity of CD38 and influence tumor cell metabolism (51). In addition, DARA can regulate the bone marrow microenvironment (52), activate immune cells, kill tumors, or directly govern the growth of tumor cells by inhibiting mitochondria (53). FDA approved it in 2015 for refractory or relapsed MM.

Isatuximab, another humanized IgG1κ mAb with a different action site from DARA, has a significant pro-apoptotic effect on tumor cells. It can effectively inhibit CD38 cyclase action, activate NK cells, and inhibit CD38-positive T regulatory cells (41). FDA approved it in March 2020 for the treatment of R/R MM. It is currently being evaluated in CD38-positive hematological malignancies for its efficacy and safety.

MOR202, a humanized CD38 antibody developed by Morphosys, is being studied as a monotherapy and combined with lenalidomide/dexamethasone or pomalidomide/dexamethasone in phase I/II trials for the treatment of refractory or relapsed MM.

TAK079, another humanized CD38 antibody(IgG1λ mAb) and TAK053, an IgG4mAb, are both in the preliminary stage

Based on the high expression of CD38 on the surface of acute leukemia cells, CD38 mAb has also gradually been applied for the treatment of acute leukemia.

### Application of CD38 mAbs in ALL

#### Preclinical studies in ALL

Studies on CD38 mAbs in B-ALL are scarce. Jutta Deckert et al. confirmed that isatuximab inhibited B-ALL cell line growth through ADCC *in vitro* (41). In a B-ALL xenograft model, isatuximab also exerted an antitumor effect, effectively prolonging mouse survival. Anlai Wang et al. reported that isatuximab exerts ADCC and ADCP effects on ALL cell lines (16 T-ALL and 11 B-ALL) and can induce robust antitumor activity *in vivo* (42).

Numerous studies have explored the efficacy of CD38 mAbs in T-ALL. It has been reported that Daratumub, Isatuximub, and other CD38 mAbs can inhibit the growth of T-All cell lines *in vitro* and exert their effects through ADCC, CDC, ADCP, and proapoptotic mechanisms (37, 41–43). In a mouse xenograft model, Naik et al. determined that DARA significantly lowered tumor load in mice compared to the control group (37). However, a patient sample (T-ALL2) mouse model manifested early death compared to the other T-ALL mouse models. The researchers hypothesized that this might be due to the high aggressiveness of the T-ALL2 patient sample. They used luciferase to label samples from this patient and then implanted the labelled samples into mice following the addition of DARA. They found no early death in mice and detected a significant reduction in fluorescence intensity. Bride et al. successfully constructed a PDX model in nonobese diabetic/severe combined immunodeficient (NOD/SCID/Il2rgtm1wjl/SzJ) mice from 15 pediatric T-ALL patients (seven ETP and eight non-ETP) (43). Daratumumab significantly reduced the leukemic burden in the bone marrow and spleen in six of the seven ETP samples compared to the control group (IgG1 mAb). Mice that received five of the eight non-ETP samples perished after the initial administration of DARA, presumably due to tumor lysis. When the tumor burden was reduced in the model, that is, following the administration of DARA, and the model was similar to the MRD-positive state, DARA exhibited significant therapeutic benefits in all eight non-ETP patient sample models compared to the control group. Both studies conducted by Naik and Bride concluded that DARA has an antitumor effect in T-ALL and can effectively eliminate MRD. Studies on its mechanism of action have identified that DARA has immune cell-mediated effects on ADCC and CDC in immune-normal mice and may promote apoptosis and inhibit CD38 enzyme activity in immune-deficient mice through nonimmune cell-mediated activities.

Regarding studies on DARA combinations, Fotini et al. demonstrated that DARA monotherapy or combined with chemotherapy can effectively improve xenograft mouse survival (54). All the mice had long-term asymptomatic survival in the DARA plus chemotherapy group. This suggests that DARA possesses remarkable antileukemia properties and may enhance the effect of chemotherapy. The researchers further assessed MRD in the bone marrow of living mice *via* PCR; seven out of eight mice were MRD-negative. These results signify that DARA also plays a decisive role in eliminating T-ALL with persistent positive MRD. P Doshi et al. described that DARA effectively inhibited the proliferation of T-ALL (P <0.05) and B-ALL (P <0.001) in animal models compared to the control group. DARA alone or combined with vincristine significantly prolonged mouse survival (> 88 days) compared to 22 days in the control group and 43 days in the vincristine monotherapy group (55).

#### Correlation between CD38 density in ALL and efficacy of CD38 mAb

The correlation between CD38 density in ALL and the efficacy of CD38 mAbs remains controversial. Jutta Deckert et al. assessed the antitumor effect of isatuximab in 15 cell lines with different CD38 expression levels (including four T-ALL and one B-ALL) and found that isatuximab induced an ADCC effect in all cell lines (41). However, its proapoptotic and CDC effects were more evident in cell lines with high CD38 expression. Similarly, Anlai Wang et al. speculated that the effects of isatuximab-mediated ADCC and ADCP were positively correlated with CD38 expression levels (42). However, Fotini did not find a correlation between CD38 expression level and DARA efficacy (54). P Doshi et al. likewise reported that DARA-mediated effects of ADCC and CDC were not directly related to CD38 expression levels (55). As previously mentioned, the antitumor effect mediated by CD38 mAb is related to the affinity between the antibody, tumor antigen, antibody, and Fc receptor. In ALL studies, distinct types of CD38mAbs were correlated with CD38 expression levels, potentially due to antibody-antigen affinity. More studies are necessary to elucidate this possibility.

#### Clinical application in ALL

Several studies concerning the efficacy and safety of DARA in ALL have been reported in recent years. Bonda et al. reported for the first time a patient with ETP-ALL who relapsed after two allogeneic hematopoietic stem cell transplantations (allo-SCTs) and multiline chemotherapy (56). Following reinduction chemotherapy, the bone marrow morphology revealed 4% blasts, while MRD reached 15.6%. Owing to the high expression of CD38 in the patient’s leukemic cells, DARA was administered. MRD was negative after four doses (16 mg/kg weekly on days 1, 8, 15, and 22). Then, the patient received DARA every two weeks for 16 weeks, along with 6-mercaptopurine and methotrexate. MRD negativity lasted for more than five months. Sumeet Mirgh et al. reported that patients with ETP were MRD-positive after chemotherapy and allo-SCT (57). Although the expression of CD38 was downregulated in residual blasts, the patient still received DARA treatment (16 mg/kg weekly for two weeks) and became MRD-negative. Yisai et al. also confirmed that in two patients with non-ETP ALL who relapsed after allo-SCT, MRD was positive after nelarabine treatment but became negative after DARA therapy (16 mg/kg dose weekly on days 1, 8, 15, and 22) (58).

T-ALL patients ineligible for standard chemotherapy, including ETP patients, can achieve MRD negativity with DARA monotherapy, followed by allo-SCT (Sumeet Mirgh et al. utilized a DARA regimen of 16 mg/kg dose weekly for eight weeks, then every two weeks for eight weeks, followed by monthly doses for two months) (57) or to reduce tumor burden before receiving standard chemotherapy (Sandra D. R used a DARA regimen of 16 mg/kg dose on days 1 and 15 for two weeks) (59). Regrettably, these two patients eventually died as a result of infection and disease relapse. These reports are also consistent with the results of preclinical studies demonstrating that CD38 mAb (DARA) has an excellent antileukemia effect in T-ALL, especially MRD-positive T-ALL. Yisai et al. reported a case of a B-ALL patient who relapsed after BITE (anti-CD3/CD19) therapy (58). Due to the high expression of CD38 in leukemia cells, DARA was added (16 mg/kg dose weekly for three weeks), and CR was achieved. Eventually, allo-SCT was performed, but the patient relapsed three months after transplantation. (**Table 1**).

**Table 1:** Case reports of CD38 targeted therapies in AL.

Author	Age	Sex	Disease	Previous treatment	Disease status before CD38 targeted therapy	Treatments	response to therapy	last follow-up
**Bonda A et al.**	32yrs	female	ETP ALL	Chemotherapy twice allo-SCT re-chemotherapy	MRD+	Daratumumab	MRD-	Sustained remission
**Sumeet Mirgh et al.**	57yrs	male	ETP ALL、Hypertension, diabetesInfection	–	Unfit for chemotherapy	Daratumumab Allo-SCT	MRD-	Die of infections
**Sumeet Mirgh et al.**	26yrs	male	ETP ALL	Chemotherapy allo-SCT	MRD+	Daratumumab	MRD-	Sustained remission for 326 days
**Yishai Ofran et al.**	44yrs	female	T-ALL	Chemotherapies Allo-SCT Re-chemotherapy	MRD+	Daratumumab	MRD-	Sustained remission for 10.5 months
**Yishai Ofran et al.**	24yrs	female	T-ALL	Chemotherapies Allo-SCT Re-chemotherapy	MRD+	Daratumumab	MRD-	Sustained remission for 10 months
**Yishai Ofran et al.**	40yrs	male	B-ALL	Chemotherapies BITEChemotherapies Allo-SCT	MRD+	Daratumumab	MRD-	Daratumumab was discontinued due to severe GVHD reaction
**Marco Cerrano**	44yrs	male	ETP ALL	Chemotherapies Allo-SCT	MRD+	Daratumumab	MRD-	Sustained remission for 3 months
**Sandra D**	2yrs	male	T-ALL	Chemotherapies	Relapsed, unfit for chemotherapy	Daratumumab	NCR	Die of T-ALL
**Guo YL et al.**	24yrs	female	B-ALL	Chemotherapy CD19/CD22 CART	Relapsed	Anti-CD38 CART	NA	died of severe CRS

A phase II trial of daratumumab combined with standard chemotherapy for relapsed and refractory B-ALL and T-ALL in children and young adults is currently underway (ClinicalTrials.gov identifier: NCT03384654). The study included B-ALL patients with two or more relapses or who had been refractory to at least two chemotherapy regimens, as were T-ALL patients relapsing for the first time or who had been refractory to one chemotherapy regimen. All patients were treated with a combination of daratumumab, prednisone, and vincristine. In the T-ALL group, doxorubicin, PEG-asparagase, cyclophosphamide, cytarabine, 6-mercaptopurine, and methotrexate were added to enhance further efficacy. Although the response rate in B-ALL patients was low, the efficacy in T-ALL patients was highly promising, with 5/8 patients achieving CR. Therefore, the study was expanded to include 20 additional T-ALL patients. It is unknown whether the poor response of CD38 mAb in B-ALL patients was attributable to the chemotherapeutic regimen or the lower expression level of CD38 in B-ALL patients compared to T-ALL patients. Another clinical trial enrolling 14 patients to evaluate the efficacy and safety of isatuximab in T-ALL (ClinicalTrials.gov identifier: NCT02999633) was prematurely terminated on November 8, 2017, due to an unsatisfactory benefit/risk ratio (**Table 2**).

**Table 2:** Clinical trials of CD38 mAbs in ALL.

Study	Phase	Disease	Setting	Treatment	Status
**NCT03860844**	I/II	Pediatric AML/ALL	refractory/relapsed	Isatuximab+Chemotherapy	Recruiting
**NCT02999633**	II	T-ALL/T-LBL	refractory/relapsed	Isatuximab	Terminated
**NCT03384654**	II	B/T Precursor Cell Lymphoblastic Leukemia/Lymphoma	refractory/relapsed	Daratumumab+Chemotherapy	Active, not recruiting
**NCT04972942**	II	T-ALL/T-LBL	high risk	Daratumumab+TBI+allo-SCT	Not yet recruiting
**NCT01084252**	I/II	CD38-positive hematologic malignancies including NHL, MM, AML, ALL, and CLL	relapsed/refractory	Isatuximab	Recruiting

### Application of CD38 mAbs in AML

#### Preclinical studies in AML

Various studies have corroborated that DARA exerts antitumor effects in AML both *in vivo* and *in vitro*. Dos et al. examined the antileukemic effect of CD38 mAb in AML cell lines and primary AML cells (38). They reported that DARA inhibited the proliferation of AML cells (MOLM-13, MOLM-16, MV-4-11, and NB4 cell lines) *in vitro*. Meike Farber et al. also found that DARA could significantly reduce AML cell growth in 5/8 AML cell lines (35). Further mechanistic studies determined that DARA induced apoptosis of AML cell lines *via* cross-linking and could induce a 5~20% ADCC effect and a 2~5% CDC effect on six of the nine AML cell lines (38). Moreover, compared with homotypic IgG1 monoclonal antibodies, DARA significantly enhanced the ADCP effect of macrophages (49, 60). Other studies have found that DARA exerts a significant antileukemic effect in coculture systems of AML cells and mesenchymal cells but only has a weak cell-autonomous effect in monoculture, suggesting that DARA is more likely to act through the bone marrow microenvironment (49). Recent studies have discovered another mechanism by which DARA limits mitochondrial transfer from mesenchymal stem cells to AML cells, thereby reducing the metabolic capacity of AML tumor cells and ultimately inhibiting tumor proliferation (53). Moreover, in a patient-derived xenograft (PDX) mouse model, DARA significantly reduced tumor burden in peripheral blood and spleen but did not affect the bone marrow (38). The authors observed that the addition of DARA did not reduce bone marrow tumor burden; nevertheless, it significantly reduced CD38 expression on the surface of bone marrow AML cells. They theorized that the bone marrow microenvironment suppressed the antileukemic effect of DARA. Another study revealed that DARA significantly reduced leukemic burden in peripheral blood compared to the control group (42%, P=0.0354), but there was no significant difference in the bone marrow (60). Last, Naik et al. demonstrated that DARA reduced tumor burden in mice compared to the control group (37).

#### Correlation between CD38 density and efficacy of DARA in AML

Earlier DARA studies in MM patients have established a positive correlation between CD38 surface density and DARA efficacy (41, 45, 46, 61). In AML studies, the relationship between the CD38 expression level and the efficacy of DARA monotherapy is complex. Meike Farber et al. noted that CD38 density seemed to be related to the efficacy of CD38 mAb (60).In contrast, Dos et al. did not observe a direct correlation between the expression level of CD38 and the effect of CD38 mAb (38). Enguerran Mouly et al. (39) determined that in THP-1 cells (low CD38 expression), the maximum ADCC effect induced by DARA was approximately 7%, whereas the maximum ADCC effect was approximately 28% in UOC-M1 cells (high CD38 expression). Furthermore, there was no significant difference in the effect of DARA-induced ADCC between the two groups of AML cell lines with low and high CD38 expression levels (Spearman’s rank correlation r=0.6134, P=0.09). After incubation with inecalcitol (a vitamin D receptor agonist enhancing CD38 expression in 10 out of 11 AML cell lines), CD38 expression levels were upregulated in both CD38-negative cell lines (HL-60, U-937) and CD38-high cell lines in varying degrees. The corresponding DARA-induced maximum ADCC effect was increased to 20% and 42%, respectively. Researchers further evinced that DARA-induced ADCC was correlated with the expression level of CD38 after incubation with inecalcitol (Spearman’s rank correlation r=0.8333, P<0.01). Furthermore, the expression level of CD38 was correlated with the ADCC effect of DARA but not with CDC. Similarly, the combination of DARA and ATRA led to a significant improvement in anti-leukemic effects, which was positively correlated with the upregulation of CD38 expression in AML cell lines (60). Collectively, these results signify that there was no significant correlation between the CD38 expression level and the effect of the CD38 mAb in leukemia. However, with an improvement in CD38 expression after stimulation by other drugs, the action of the CD38 mAb is further amplified. The antitumor effect mediated by CD38 mAb is linked to various factors, including the affinity between the antibody and tumor antigen, the affinity between the antibody and Fc receptor, the density of tumor antigen, the characteristics of the tumor target cells, and the characteristics of the immune effector cell. The expression level of CD38 in AML cells did not correlate with the efficacy of CD38 mAb alone but rather with the efficacy of combination therapy. We infer that this may be attributed to the lower expression level of CD38 in AML cells than in MM cells, which is insufficient to observe the differences in antitumor effects of CD38 mAb in AML cells with varying CD38 expression levels. However, the combination with other drugs significantly increases the CD38 expression level in AML cells, reaching the threshold to observe the efficacy of CD38 mAb. Nonetheless, whether this observation is also related to the features of AML cells remains to be determined.

#### DARA-based combination for AML

The above studies provide a theoretical foundation for a DARA-based combination that may enhance antitumor activity.

Prior studies have identified that ATRA can upregulate CD38 expression in various tumor cells, including APL (62) and MM (46). Yoshida et al. reported that ATRA upregulated CD38 expression in KG-1, U937, other AML cell lines, and primary cells of patients (61). Correspondingly, Buteyn et al. demonstrated that ATRA upregulated CD38 expression in AML cell lines (MV4-11, OCIAML3, MOLM-13, and U937) and primary cells of AML patients (63). Thus, there is a binding site of the ATRA receptor RAR (retinoic acid receptor) in the first intron of the CD38 gene, directly activating RAR and initiating CD38 transcription after ATRA binding (12). The increased expression of CD38 can improve the antibody-mediated cytotoxicity of DARA and the anti-leukemic effect. Additionally, Buteyn et al. reported that ATRA combined with DARA improved conjugate formation and the antibody-mediated cytotoxicity of primary AML cells through FCR induction *in vitro* compared to the control and DARA monotherapy groups. The survival of AML cell lines and primary cells was significantly reduced at 24, 48, and 72 h. *In vivo*, ATRA with DARA inhibited tumor proliferation in mice, significantly decreased tumor size, and prolonged survival compared to DARA monotherapy (63).

Venetoclax, a Bcl-2 inhibitor, also showed a synergetic effect with DARA. It is FDA-approved for AML combined with low-dose chemotherapy or demethylation. However, its extensive use eventually leads to resistance, making its combination with other drugs essential. According to some reports, mitochondria can be transferred from mesenchymal cells to primary AML cells to promote AML growth. DARA has been reported to inhibit mitochondrial be transferred from mesenchymal cells to primary AML cells (53). Mistry et al. (64) determined that venetoclax monotherapy inhibited AML cell growth and promoted apoptosis in primary AML cells and mesenchymal cell cocultures, whereas DARA monotherapy did not. Compared to venetoclax and DARA monotherapies, combination therapy inhibited tumor cell growth and promoted apoptosis. *In vivo*, they also demonstrated that venetoclax combined with DARA effectively reduced tumor burden in mice.

In addition, Fatehchand et al. (65). reported that IFN-γ promoted the differentiation of primary AML cells and upregulated CD38 and FcγRI expression. *In vitro*, IFN-γ enhanced the antibody-mediated cytotoxicity of DARA, while IFN-γ combined with DARA inhibited tumor growth in animals.

#### Clinical trials of CD38 mAbs in AML

Preclinical studies have corroborated the efficacy of CD38 mAbs in AML, and DARA-related clinical trials in AML are underway. Researchers at the MD Anderson Cancer Center evaluated the efficacy of daratumumab in patients with refractory relapsed AML and high-risk MDS (ClinicalTrials.gov identifier: NCT03067571). A phase I/II trial of daratumumab and DLI (donor lymphocyte infusions) in patients with relapsed AML after hematopoietic stem cell transplantation (ClinicalTrials.gov identifier: NCT03537599) is ongoing at Ohio State University. Another CD38 mAb, isatuximab, is also being tested in phase I/II clinical trials combined with chemotherapy for refractory relapsed AML and ALL in children (ClinicalTrials.gov identifier: NCT03860844). The outcomes of these clinical trials are not yet available (**Table 3**).

**Table 3:** Clinical trials of CD38 mAbs in AML.

Study	Phase	Disease	Setting	Treatment	Status
**NCT03067571**	I/II	AML and high-risk MDS	refractory/relapsed	daratumumab	Unknown
**NCT03537599**	I/II	AML with allo-SCT	relapsed	Daratumumab+DLI	Recruiting
**NCT03860844**	I/II	Pediatric AML/ALL	refractory/relapsed	Isatuximab+Chemotherapy	Recruiting
**NCT01084252**	I/II	CD38-positive hematologic malignancies including NHL, MM, AML, ALL, and CLL	refractory/relapsed	Isatuximab	

## Anti-CD38 bispecific antibody

Anti-CD38/CD3 bispecific antibodies, including AMG 424, GBR1342, and Bi38-3, have been shown to be effective in MM both *in vitro* and *in vivo* (66, 67). CD38 bispecific antibodies can bind to CD38 on tumor cells and cytotoxic immune effector cells (T cells, a small number of NK cells), playing the antitumor role of the antibody itself and activating effector cells to attack tumor cells. However, these antibodies have not been explored in AL.

XmAb18968, a novel anti-CD38/CD3 bispecific antibody, binds to CD38 on the cell surface *via* the FC segment and nonselectively activates effector T cells (68). The researchers optimized XmAb18968’s affinity for CD38 and CD3 to lower cytokine release. A phase I clinical trial of XmAb18968 (NCT05038644) will be conducted to evaluate its safety and tolerability in patients with relapsed and refractory T-ALL, T-LBL, and AML. The inclusion criteria are as follows: patients older than 18 years, having CD38 expression levels greater than 20%, and having not undergone allo-SCT within the past six months. The study’s primary endpoint is to determine the recommended dose of the phase II study and the toxicity profile, while the secondary endpoints include response rate, response duration, and survival. The study will begin to enroll patients in January 2022.

## Anti-CD38 CAR-T cells therapy

Adoptive cell therapy has achieved favorable success in B-ALL, but complications such as late B cell failure still exist, and its application in AML and T-ALL requires further exploration. Therefore, it is critical to identify new therapeutic targets for AML and ALL cell immunotherapy. As pointed out earlier, CD38 can serve as a new target for adoptive cell therapy of AML and ALL.

### Preclinical studies of CD38 CAR-T cells in AL

Esther Drent et al. designed anti-CD38 CAR-T cells using different CD38 antibody sequences and assessed their efficacy and safety (69). They found that anti-CD38 CAR-T cells were effective in lysing primary cells from AML patients, regardless of whether these cells had a high or low expression of CD38. Yoshida et al. reported that anti-CD38 CAR-T cells displayed time- and quantity-dependent cytotoxicity to AML cell lines with high expression of CD38 and had a selective killing effect on AML cell lines with partial or low expression of CD38 but had no effect on CD38-negative cells (70). However, these cytotoxic effects of anti-CD38 CAR-T cells alone on AML cell lines and primary AML cells were limited. Anti-CD38 CAR-T cells exhibited significantly enhanced antitumor activity when AML cells were cocultured with ATRA.

### Clinical studies of CD38 CAR-T cells in AL

The preclinical studies provided a theoretical foundation for the clinical application of anti-CD38 CAR-T cells in AL. Guo et al. reported a case of relapsed B-All treated with anti-CD38 CAR-T cells (71). It was a 24-year-old relapsed and refractory B-ALL patient who had received bispecific CD19/CD22 CAR-T therapy and achieved CR but relapsed five months later. At that time, CD19 was negative, and CD22 was marginally positive, but the positive rate of CD38 was 63%. Therefore, the patient was treated with anti-CD38 CAR-T cells (1×10^6^/kg). After treatment, the tumor burden in the patient’s bone marrow and peripheral blood was significantly reduced (14.52% vs. 0.8%, and 5% vs. 0%, respectively). However, the patient suffered from a severe CRS reaction combined with liver and lung toxicity (**Table 4**). Moreover, CD38 was expressed in CAR-T cells, leading to CAR-T cell death and loss of function. Finally, the patient discontinued all treatments. This case shows that anti-CD38 CAR-T cell therapy may be effective in patients with relapsed ALL even after anti-CD19 CAR-T cell therapy. Can the combination of CAR-T cell therapy with different targets improve therapeutic effects? Relevant clinical studies are currently underway.

**Table 4:** Clinical trials of CD38 CAR-T cell therapies in AL.

Study	Phase	Disease	Setting	Treatment	Status
**NCT03754764**	I/II	B-ALL	refractory/relapsed, after CD19 CART	Anti-CD38 CART	Recruiting
**NCT04016129**	I/II	CD19 negative ALL	refractory/relapsed	Anti-CD38 CART	Recruiting
**NCT03222674**	I/II	AML	refractory/relapsed	Muc1/CLL1/CD33/CD38/CD56/CD123 CART	Unknown
**NCT03473457**	I/II	AML	refractory/relapsed	CD38/CD33/CD56/CD123/CD117CD133/CD34/Mucl CART	Terminated
**NCT04351022**	I/II	AML	relapsed/refractory	Anti-CD38 CART	Recruiting

Han et al. conducted a phase I/II study (NCT03754764) to assess the safety and feasibility of anti-CD38 CAR-T cell therapy in B-ALL patients who had previously received CD19 CAR-T therapy. There is also a phase I/II study to evaluate the safety and efficacy of CD38 CAR-T therapy in CD19-negative ALL patients, particularly in ALL patients treated with CD19 CAR-T cells (NCT04016129). Chang et al. performed a phase I/II study of multitarget CAR-T cells, including CD38, in refractory relapsed AML patients to evaluate the feasibility, safety, and efficacy of treatment (NCT03222674). Another similar study is ongoing (NCT03473457) (**Table 4**). In another study, Cui et al. evaluated the efficacy and safety of anti-CD38 CAR-T cells in patients with relapsed AML following allogeneic hematopoietic stem cell transplantation (NCT04351022) (72). The study included six patients with relapsed posttransplant AML with a median pre-treatment CD38 positive rate of 95% (92–99%). All patients received FC regimens (fludarabine and cyclophosphamide) prior to CD38 CART cell infusion (6.1-10×10^6^/kg). Four weeks after anti-CD38 CAR-T cell infusion, 4/6 patients (66.7%) achieved CR or CRi, with a median time to CR or CRi of 191 days, a median OS of 7.9 months, and a median leukemia-free survival (LFS) of 6.4 months. Five patients experienced grade 1-2 CRS, and one developed grade 3 hepatic toxicities.

These findings provide evidence of the effectiveness of anti-CD38 CAR-T cells in the treatment of AL. However, owing to various adverse reactions, it is vital to explore new approaches for mitigating adverse reactions and prolonging the time of action *in vivo*. Presently, some researchers are attempting to employ proteins or antibodies to block CD38 on CAR-T cells, knock out the CD38 gene in effector cells, and use the caspase-9 suicide gene to mediate the autolysis of CAR-T cells.

## Conclusions

Targeting CD38 antibodies and cellular therapies have demonstrated a unique and encouraging therapeutic effect in MM. They have also exhibited promising results in AL, especially in T-ALL. Combining CD38 mAb with ATRA, venetoclax, or IFN-γ for AML are being explored in clinical trials and real-world studies to validate the efficacy. The correlation between CD38 density and the efficacy of CD38 mAb in AL remains controversial and warrants further study. In the future, screening eligible predictors will assist in stratifying AL patients who can benefit from CD38-targeted therapies. Additional studies are needed to elucidate the efficacy and safety of CD38-targeted therapies, such as bispecific antibodies, trispecific antibodies, ADCs, multitarget CARTs, and CAR-NK cell therapy, in acute leukemia.

## Author contributions

XZ: Conceptualization, literature retrieval and utilization, writing-original draft. HM: Conceptualization, supervision, writing-review and editing This manuscript is approved by all authors for publication.

## Conflict of interest

The authors declare that the research was conducted in the absence of any commercial or financial relationships that could be construed as a potential conflict of interest.

## Publisher’s note

All claims expressed in this article are solely those of the authors and do not necessarily represent those of their affiliated organizations, or those of the publisher, the editors and the reviewers. Any product that may be evaluated in this article, or claim that may be made by its manufacturer, is not guaranteed or endorsed by the publisher.
